# Relationship Between Fermented Food Consumption Patterns, hs-CRP, and Chronic Diseases Among Middle-Aged Koreans: Data from the 2015–2018 Korea National Health and Nutrition Examination

**DOI:** 10.3390/nu17081343

**Published:** 2025-04-14

**Authors:** Sori On, Woori Na, Cheongmin Sohn

**Affiliations:** 1 Department of Food and Nutrition, Wonkwang University, 460 Iksan-daero, Iksan, Jeonbuk 54538, Republic of Korea; onsori000803@gmail.com (S.O.); nawoori6@gmail.com (W.N.); 2 Institute of Life Science and Natural Resources, Wonkwang University, Iksan, Jeonbuk 54538, Republic of Korea

**Keywords:** fermented foods, dietary patterns, inflammation, high-sensitivity c-reactive protein, chronic diseases

## Abstract

**Background/Objectives**: Fermented foods promote digestion and may help prevent chronic diseases. However, studies on their relationship with health indicators in Korea remain limited. This study aimed to identify fermented food consumption patterns among middle-aged Korean adults and to analyze their association with high-sensitivity C-reactive protein (hs-CRP) and chronic disease. **Methods**: This study analyzed 7111 adults aged 40–64 years from the 6th–7th Korea National Health and Nutrition Examination Survey (KNHANES, 2015–2018). hs-CRP values were categorized as <1 mg/L, 1–3 mg/L, and ≥3 mg/L. Latent profile analysis (LPA) classified fermented foods into 10 categories using Mplus 8.11, with LMR-LRT significance and entropy ≥ 0.7 determining the number of classes. Logistic regression analysis using SPSS 29.0 was conducted to calculate odds ratios (ORs) and 95% confidence intervals (CIs) for hs-CRP and chronic disease (*p* < 0.05). **Results**: LPA identified four consumption patterns: <Low Fermented-Food Pattern> (LFP), <Fermented Alcohol- and Beverage-Centered Pattern> (FABP), <Fermented Dairy-Centered Pattern> (FDP), and <Fermented Grain-Centered Pattern> (FGP). hs-CRP was highest in LFP (1.0 ± 1.2 mg/L), followed by FABP (0.8 ± 1.1 mg/L) and FDP and FGP (0.9 ± 1.2 mg/L) (*p* < 0.001). Compared to LFP, FGP had ORs of 0.810 (95% CI: 0.690–0.950, *p* < 0.005) for hypertension and 0.586 (95% CI: 0.459–0.747, *p* < 0.001) for diabetes. For dyslipidemia, ORs were 0.832 (95% CI: 0.720–0.962, *p* < 0.005) for FABP and 0.832 (95% CI: 0.719–0.962, *p* < 0.005) for FDP. **Conclusions**: This study classified fermented food consumption patterns and analyzed their association with hs-CRP and chronic disease. FGP and FDP showed lower inflammation and reduced odds of hypertension, diabetes, and dyslipidemia compared to LFP. These findings highlight the potential of healthy fermented food consumption patterns to support inflammation control and chronic disease prevention.

## 1. Introduction

Fermented foods are traditional foods that have developed over a long period across the world, evolving uniquely in different regions with their own local ingredients and methods [1]. These fermented foods have long been used as a way to preserve food for long periods of time and are known to have beneficial effects on human health, including improved digestion, improved gut health, strengthening of the immune system, and preventing chronic diseases. Fermentation is a process by which microorganisms, especially beneficial ones, such as probiotics, utilize carbohydrates to produce fermentation products, such as alcohol, organic acids, and CO_2_ [2,3]. The acids and alcohols generated during fermentation contribute to inhibiting the growth of pathogenic bacteria in food [4]. These beneficial effects of fermentation are well exemplified by traditional Korean fermented foods. In Korea, representative fermented foods include kimchi, soy-based fermented products, such as ganjang (soy sauce), doenjang (fermented soybean paste), gochjang (fermented red pepper soybean paste), and cheonggukjang (fast-fermented soybean paste), as well as jeotgal (fermented seafood) and makgeolli (fermented rice wine). These foods have been reported to have positive effects on health. Compared to a non-fermented diet, diets containing fermented foods have been associated with an increase in gut microbial diversity and a higher proportion of beneficial bacteria [5]. Fermented foods contain beneficial microorganisms that regulate gut microbiota balance and promote gut health through their metabolic products and bioactive compounds. Lactic acid bacteria and fermentation-derived metabolites found in various fermented foods have been reported to promote the growth of beneficial bacteria, compete with pathogenic bacteria in the gut, and perform immunomodulatory functions. The conversion of bioactive peptides and phenolic compounds produced during fermentation may contribute to immune and metabolic health [6]. The consumption of various fermented foods has also been linked to the prevention of chronic diseases [7].

Middle age is a period during which individuals become more vulnerable to chronic diseases due to physiological changes. According to research, approximately 34.4% of middle-aged women aged 40–64 have at least one chronic disease [8]. Among individuals in this age group, dyslipidemia, hypertension, and diabetes have been identified as major chronic diseases [9]. Additionally, data from Statistics Korea in 2022 indicate that hypercholesterolemia, diabetes, and hypertension continue to increase with age among individuals aged 40 to 69 [10,11,12]. Some studies have suggested that fermented foods may be beneficial in preventing chronic diseases, suggesting that incorporating fermented foods into dietary habits is important for health management in middle-aged adults.

Studies have reported that the consumption of fermented soybean protein can have a positive effect on blood pressure reduction [13], and fermented soybeans fortified with *Enterococcus faecium* and *Lactobacillus jugurti* have been shown to reduce total cholesterol and triglyceride (TG) levels and increase high-density lipoprotein (HDL) cholesterol levels in a high-cholesterol dietary setting, suggesting that long-term consumption may contribute to the improvement of dyslipidemia [14]. Furthermore, an increase in kimchi consumption has been associated with a decrease in serum cholesterol and low-density lipoprotein (LDL) cholesterol levels, indicating that fermented foods may also play a role in preventing lipid-related diseases [15]. Research has also shown that the long-term consumption of fermented soybean extract is effective in reducing glycated hemoglobin (HbA1c) levels in individuals with mild type 2 diabetes [16]. High-sensitivity C-reactive protein (hs-CRP) is one of the key biomarkers used to predict the risk of diseases related to chronic inflammation. It is a high-sensitivity quantitative assay developed to measure CRP with greater precision. Elevated hs-CRP levels indicate increased systemic inflammation, which has been associated with a higher risk of various chronic diseases, including cardiovascular conditions. It serves as a useful biomarker for assessing the overall inflammatory status of the body [17]. Some studies have shown that meju and fermented soybean fiber extracted from meju have a CRP-lowering effect [18]. Additionally, the consumption of cheonggukjang has been suggested to reduce hs-CRP, indicating a potential anti-inflammatory effect [19].

Recent studies have increasingly focused on analyzing the relationship between diet and health by examining overall dietary patterns rather than individual food items [20,21,22]. Therefore, analyzing the relationship between fermented foods, chronic diseases, and hs-CRP at the dietary pattern level, rather than at the individual food level, may yield more meaningful insights. Latent profile analysis (LPA) is an analytical method that identifies patterns of fermented food consumption based on actual data without assuming predefined patterns. This approach is useful for classifying groups according to consumption patterns rather than individual food items.

The aforementioned research findings indicate that fermented foods go beyond the category of traditional cuisine and have become essential dietary components for health promotion and the prevention of chronic diseases. However, in Korea, studies analyzing the relationship between fermented food consumption patterns and health indicators remain limited. Therefore, this study aims to classify fermented food consumption patterns among middle-aged adults in Korea using LPA and analyze how each type is related to hypertension, diabetes, dyslipidemia, and hs-CRP.

## 2. Materials and Methods

### 2.1. Study Population

**Figure 1:**
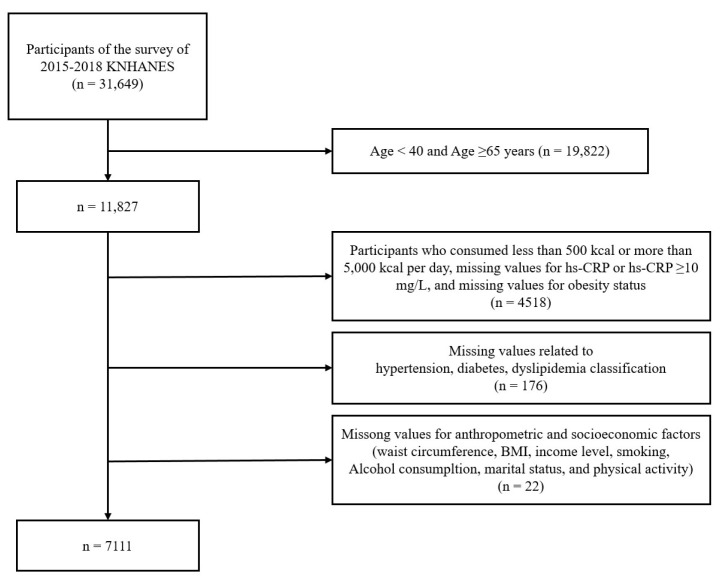
Flow chart representing the selection of study participants. KNHANES, Korea National Health and Nutrition Examination Survey; hs-CRP, high sensitivity C-reactive protein; BMI, body mass index.

This study analyzed raw data from the 6th and 7th (2015–2018) Korea National Health and Nutrition Examination Survey (KNHANES). Among a total of 31, 649 participants, 11,827 adults aged 40 to 64 years were included. Participants were excluded if they met the following criteria: those with an energy intake of less than 500 kcal or more than 5000 kcal per day (n = 1758), those with missing data for the hs-CRP variable (n = 1002), those with hs-CRP of 10 mg/L or higher (n = 105), those with missing data on obesity status (n = 1653), and those with missing data for variables related to hypertension, diabetes, dyslipidemia classification, waist circumference, income, body mass index (BMI), smoking status, alcohol consumption, marital status, and physical activity (n = 198). After applying these exclusion criteria, a total of 7111 participants (n = 2765 men, n = 4346 women) were included in the final analysis. The selection process for this study was as follows (Figure 1). The present study was conducted in accordance with the principles of the Declaration of Helsinki (1975, revised in 2013). Ethical approval was obtained from the Clinical Test Deliberation Commission of the Institutional Review Board at Wonkwang University, Iksan City, Korea (Approval No. WKIRB-202412-SB-088).

### 2.2. Classification of Fermented Foods

In this study, fermented foods were classified into the following 10 categories: grains, fermented soybean products, vinegar, vegetables, fish and seafood, fruits, dairy products, alcoholic beverages, sauces, and leaf teas and beverages. This classification was based on multiple references while considering the cultural characteristics of Korean dietary habits to establish a Korea-specific categorization [23,24,25,26,27].

Grain-based fermented foods included fermented bread and traditional Korean fermented rice cakes, such as jeungpyeon. Fermented soybean products (jang) included ganjang (soy sauce), doenjang (fermented soybean paste), gochjang (fermented red pepper soybean paste), cheonggukjang (fast-fermented soybean paste), ssamjang (a mixture of gochujang and doenjang), and chunjang (fermented black soybean paste). Vinegar products included both regular vinegar and vinegar-based beverages. The vegetable category included pickled vegetables, such as danmuji (yellow pickled radish), jangajji (korean pickled vegetables), and various types of kimchi. Fish and seafood included fermented seafood, such as jeotgal (fermented seafood), aekjeot (fermented fish sauce), and fermented fish. Fermented fruit products included fermented fruits, such as olives and plums. The dairy category included fermented milk and cheese. Alcoholic beverages included fermented alcoholic drinks, such as beer, wine, makgeolli (Korean rice wine), fruit wines, and cheongju (korean refined rice wine). The sauce category included balsamic dressing and hot sauce. Finally, the fermented tea and beverage category included fermented teas and fermented drinks. Details of the foods included in each fermented food group are shown in Table S1.

### 2.3. hs-CRP and Disease Classification

In this study, hs-CRP values were categorized based on risk levels as follows: less than 1 mg/L was classified as low risk, 1–3 mg/L was classified as average risk, and 3 mg/L or higher was classified as high risk [28]. The diagnostic criteria for diabetes, hypertension, and dyslipidemia were defined as follows. The diagnosis of diabetes was based on the criteria established by the American Diabetes Association (ADA) [29]. Individuals were classified as having diabetes if they met any of the following conditions: FBG level of 126 mg/dL or higher, HbA1c level of 6.5% or higher, a diagnosis of diabetes by a physician, insulin injection treatment, or the use of diabetes medication. The classification of hypertension followed the guidelines of the Korean Society of Hypertension (KSH) [30]. Individuals were classified as having hypertension if they met any of the following criteria: a systolic blood pressure of 140 mmHg or higher, a diastolic blood pressure of 90 mmHg or higher, a diagnosis of hypertension by a physician, or the use of antihypertensive medication. The diagnosis of dyslipidemia followed the criteria established by the National Cholesterol Education Program (NCEP) Adult Treatment Panel III (ATP III) [31]. Dyslipidemia was identified if any of the following conditions were met: HDL cholesterol level below 40 mg/dL, total cholesterol level of 240 mg/dL or higher, TG level of 200 mg/dL or higher, LDL cholesterol level of 160 mg/dL or higher, a diagnosis of dyslipidemia by a physician, or the use of lipid-lowering medication. LDL cholesterol levels were calculated using the Friedewald formula.

### 2.4. General Characteristics

The general characteristics included sociodemographic and health behavior factors. Sociodemographic factors consisted of sex, age, income level, and marital status. Income level was categorized into four groups: “low”, “lower middle”, “upper middle”, and “high”. Marital status was classified as either “married” or “unmarried”. Health behavior factors included body mass index (BMI), smoking status, alcohol consumption, and physical activity. Alcohol consumption was categorized as “yes” or “no”, while smoking status was classified as “yes” or “no”. Physical activity status was determined based on engagement in moderate-intensity physical activity and was categorized as “yes” or “no”.

### 2.5. Statistical Analysis

In this study, latent profile analysis (LPA) was conducted using the zero-inflated Poisson (ZIP) model in Mplus version 8.11 (Muthén & Muthén, Los Angeles, CA, USA). Before applying the ZIP model, natural log transformation was performed based on the distribution of the data, and the data were rounded to meet the model’s requirement for integer values. The ZIP model, which is suitable for datasets with a high proportion of zeros [32,33], was used to classify latent profiles based on fermented food consumption and to calculate the adjusted means for each profile. The number of latent profiles progressively increased from one to five, and model fit was evaluated using the Akaike information criterion (AIC), Bayesian information criterion (BIC), and adjusted BIC (aBIC), with lower values indicating better fit. Entropy values were used to assess classification quality, and the Lo–Mendell–Rubin (LMR) test and the bootstrap likelihood ratio test (BLRT) were performed to determine model significance, with *p*-values < 0.05 considered statistically significant. All other statistical analyses, including the examination of relationships between latent profiles and health outcomes, were conducted using SPSS version 29.0 (IBM Corp., Armonk, NY, USA), with the significance level set at 5%. Chi-square tests were conducted to analyze the relationship between fermented food consumption patterns and hs-CRP, while logistic regression analysis was used to calculate odds ratios (ORs) and confidence intervals (CIs) for the association between fermented food consumption patterns and diseases. Additionally, chi-square tests and ANOVA were performed to compare demographic characteristics among the latent profiles and to analyze general differences in characteristics based on fermented food consumption patterns.

## 3. Results

### 3.1. Latent Profile Analysis (LPA)

**Figure 2:**
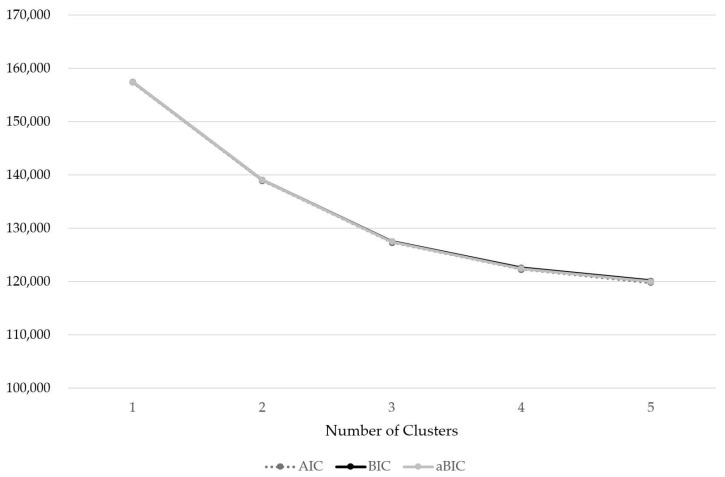
Latent profile analysis (LPA).

As a result of the LPA, the most appropriate model was determined to be the four-pattern model (Table 1 and Figure 2). The model fit analysis showed that the Lo–Mendell–Rubin likelihood ratio test (LMR-LRT) values were statistically significant across all models, and the entropy values were above 0.7 in all models, indicating a relatively accurate classification. In this study, the final model was selected based on the highest entropy value and the lowest values for AIC, BIC, and aBIC, which assess model fit.

### 3.2. Fermented Food Consumption Patterns

The names of the clusters were determined based on the adjusted mean intake of each cluster. Fermented foods, such as fermented soybean products (jang) and kimchi, as well as vegetables, are commonly and consistently consumed across the Korean population. Given their limited discriminative power among clusters, these food groups were not reflected in the naming of the clusters. Accordingly, four dietary patterns were identified and labeled as follows: <Low Fermented-Food Pattern> (LFP), <Fermented Alcohol- and Beverage-Centered Pattern> (FABP), <Fermented Dairy-Centered Pattern> (FDP), and <Fermented Grain-Centered Pattern> (FGP) (Table 2).

(1) Cluster I (low fermented-food pattern, LFP): Middle-aged adults in this group consumed very little fermented food overall.
(2) Cluster II (fermented alcohol- and beverage-centered Pattern, FABP): This group primarily consumed fermented alcoholic beverages, such as beer, wine, and makgeolli.
(3) Cluster III (fermented dairy-centered pattern, FDP): This group predominantly consumed fermented dairy products, such as fermented milk and cheese.
(4) Cluster IV (fermented grain-centered pattern, FGP): This group mainly consumed fermented grain-based foods, such as bread and traditional Korean rice cakes, as well as fermented dairy products.

### 3.3. General Characteristics by Fermented Food Consumption Patterns

The general characteristics according to fermented food consumption patterns are shown in Table 3. Among the 7111 participants, 2765 were men and 4346 were women. A comparison of sex distribution across the 4 consumption patterns revealed that the LFP group (2309 participants, 59.9%), FDP group (701 participants, 70.2%), and FGP group (796 participants, 69.2%) had a higher proportion of women. In contrast, the FABP group had a higher proportion of men (565 participants, 51.1%) (*p* < 0.001). A significant difference in BMI was observed among the groups (*p* < 0.001). The LFP group had an average BMI of 23.5 ± 3.0 kg/m^2^, while the FABP group showed a BMI of 23.4 ± 2.9 kg/m^2^. The FDP group had an average BMI of 23.1 ± 2.9 kg/m^2^, and the FGP group had the lowest BMI at 23.0 ± 2.9 kg/m^2^ (*p* < 0.001). hs-CRP concentrations showed significant differences among the groups, with the LFP group at 1.0 ± 1.2 mg/L, the FABP group at 0.9 ± 1.1 mg/L, and both the FDP and FGP groups at 0.9 ± 1.2 mg/L (*p* < 0.001). The nutrient intake according to fermented food consumption patterns is shown in Table 4. The energy intake of the LFP group was the lowest at 1846.7 ± 732.6 kcal, while the FABP group had the highest intake at 2407.8 ± 905.9 kcal, showing a significant difference between the groups (*p* < 0.001). In terms of carbohydrate intake, the LFP group consumed 297.1 ± 118.7 g, whereas the FGP group had the highest intake at 323.6 ± 123.9 g (*p* < 0.001).

### 3.4. Relationship Between Fermented Food Consumption Patterns and hs-CRP

The results of the chi-square test on the association between fermented food consumption patterns and hs-CRP are presented in Table 5. Among participants with an hs-CRP below 1 mg/L, the FDP group had the highest number of individuals (788 participants, 79.0%), whereas the LFP group had the lowest number (2844 participants, 73.7%). Among participants with an hs-CRP of 3 mg/L or higher, the LFP group had the highest number of individuals (252 participants, 6.5%), while the FDP group had the lowest (49 participants, 4.9%) (*p* < 0.005). The results of the logistic regression analysis between fermented food consumption patterns and hs-CRP are shown in Table 6. After adjusting for sex, age, current smoking status, and alcohol consumption, when compared to the LFP group, the OR for the FABP group in the 1–3 mg/L hs-CRP range was 0.804 (95% CI: 0.665–0.971, *p* < 0.005), while no significant differences were observed for the FDP and FGP groups. Additionally, in the 3 mg/L or higher hs-CRP group, the OR for the FDP group was 0.724 (95% CI: 0.529–0.991, *p* < 0.005), whereas no significant differences were observed for the FABP and FGP groups.

### 3.5. Analysis of Odds Ratios for Diseases by Fermented Food Consumption Patterns

The analysis of ORs for diseases according to fermented food consumption patterns is presented in Table 7. After adjusting for sex, age, current smoking status, and alcohol consumption, comparisons were made using the LFP group as the reference group. For hypertension, the FGP group had a significantly lower OR of 0.810 (95% CI: 0.690–0.950, *p* < 0.005) compared to the LFP group, while the FABP group had an OR of 0.971 (95% CI: 0.829–1.139), which was not statistically significant. For diabetes, the FGP group had an OR of 0.586 (95% CI: 0.459–0.747, *p* < 0.001), and the FDP group had an OR of 0.621 (95% CI: 0.486–0.794, *p* < 0.001), both showing statistically significant differences. The FABP group also showed a significantly lower OR of 0.760 (95% CI: 0.606–0.951, *p* < 0.005). For dyslipidemia, both the FDP and FABP groups had significantly lower ORs of 0.832 (FDP: 95% CI: 0.719–0.962, *p* < 0.005; FABP: 95% CI: 0.720–0.962, *p* < 0.005), whereas the FGP group did not show a statistically significant difference.

## 4. Discussion

In this study, we classified fermented food consumption patterns among middle-aged adults aged 40–64 years who participated in the 6th and 7th KNHANES (2015–2018) using LPA. We further analyzed the relationship between fermented food consumption patterns and hs-CRP, hypertension, diabetes, and dyslipidemia. Fermented food consumption patterns were categorized into four groups, namely LFP, FABP, FDP, and FGP, showing significant differences in hs-CRP and chronic diseases across the different consumption patterns.

After adjusting for age, sex, alcohol consumption, and smoking status, the relationship between fermented food consumption patterns and hs-CRP was analyzed. As a result, compared to the hs-CRP values below 1 mg/L, in the 1–3 mg/L range, the FDP group showed a significantly lower hs-CRP than the LFP group. Although these groups had lower odds ratios, the results were not statistically significant. Similarly, compared to the hs-CRP values below 1 mg/L, in the 3 mg/L or higher range, the FABP group showed a significantly lower hs-CRP than the LFP group. While lower odds ratios were also observed in the FGP and FDP groups, the results were not statistically significant. Fermented foods may have a positive effect on lowering inflammatory markers, such as hs-CRP, as they help regulate gut microbiota balance and contain bioactive compounds with anti-inflammatory properties [34]. In particular, fermented foods are known to contain beneficial probiotics, which can modulate gut microbiota composition and help reduce inflammation levels [35].

The analysis of hs-CRP distribution according to fermented food consumption patterns showed that the FDP group had the highest proportion of individuals with hs-CRP values below 1 mg/L and the lowest proportion of individuals with hs-CRP values of 3 mg/L or higher. Major lactic acid bacteria found in dairy products, such as *Lactobacillus helveticus*, *Streptococcus thermophilus*, *Lactococcus lactis*, *Propionibacterium freudenreichii*, and *Lactobacillus delbrueckii* ssp., along with their metabolites, including exopolysaccharides, peptides, lactic acid, vitamin K_2_, vitamin B_2_, and 1,4-dihydroxy-2-naphthoic acid, have been reported to contribute to anti-inflammatory effects and intestinal mucosal protection [36,37]. These physiological effects may have indirectly contributed to the reduction in hs-CRP, suggesting that the FDP group might have exhibited relatively lower hs-CRP. Meanwhile, in the FGP group, the proportion of individuals with hs-CRP of 3 mg/L or higher was higher than those in the 1–3 mg/L range. This may be attributed to the higher carbohydrate intake among individuals in this group compared to other groups and may suggest a tendency that aligns with previous research findings indicating a positive association between increased carbohydrate intake and higher hs-CRP [38]. Therefore, higher carbohydrate consumption may have contributed to the elevation of hs-CRP. Although the absolute differences in hs-CRP levels may appear small, transitions across clinical categories, such as from ≥3 mg/L to 1–3 mg/L or from 1–3 mg/L to <1 mg/L, are considered clinically meaningful. Such shifts reflect reductions in systemic inflammation and are associated with a decreased risk of various chronic diseases, including cardiovascular conditions [39]. In this study, the distribution of hs-CRP categories varied by fermented food consumption patterns. In particular, the FDP and FABP groups showed a tendency toward lower-risk hs-CRP categories. These findings may carry clinical implications beyond statistical significance. Specifically, the FDP group may lower hs-CRP through the anti-inflammatory effects of probiotics and their metabolites. In contrast, the FGP group, characterized by higher carbohydrate intake, may contribute to elevated inflammation levels, as supported by previous studies linking high carbohydrate consumption with increased hs-CRP.

The analysis of general characteristics according to fermented food consumption patterns showed that the FABP group had the highest HDL cholesterol concentration, the lowest LDL cholesterol concentration, and the lowest proportion of individuals with a hs-CRP value of 3 mg/L or higher. The average age of this group was 50.7 ± 7.1 years, the lowest among all groups, which may suggest that the relatively younger age of the participants contributed to better blood lipid profiles and lower hs-CRP.

This study confirmed that the ORs for chronic diseases varied by fermented food consumption patterns, aligning with previous findings that fermented food intake has a beneficial effect in reducing chronic disease [40]. After adjusting for age, sex, alcohol consumption, and smoking status, the analysis of the relationship between fermented food consumption patterns and chronic diseases showed that compared to LFP, FGP had significantly lower rates of hypertension (*p* < 0.001) and diabetes (*p* < 0.001). Moreover, FDP and FABP had significantly lower rates of diabetes (*p* < 0.001) and dyslipidemia (*p* < 0.001) than LFP. These findings suggest that fermented food consumption may help reduce hs-CRP and lower the risk of chronic diseases. Despite adjustments for sex, age, smoking, drinking, marital, income, physical activity, and energy, the results did not change substantially. This indicates that the relationship between fermented food consumption patterns and hs-CRP, as well as chronic diseases, is robust.

Additionally, the FGP group tended to be associated with a lower risk of hypertension and diabetes, while the FDP and FABP groups were linked to a lower risk of diabetes and dyslipidemia. This suggests that different types of fermented foods may have distinct health benefits. Meanwhile, the FDP and FGP groups showed relatively lower rates of alcohol consumption and smoking, indicating that the lifestyle habits of individuals in these groups may be associated with health-conscious dietary patterns [41]. As the results of this study suggest that fermented food consumption patterns are associated with lower hs-CRP and reduced chronic disease risk, it is likely that fermented food intake plays a key role in these health benefits, with lifestyle habits acting as complementary factors. Therefore, strategies for promoting fermented food consumption while considering lifestyle factors should be considered for the prevention of chronic diseases.

Across all patterns, the adjusted mean intake of fermented soybean products and vegetables was high. This suggests that the consumption of fermented vegetables, such as fermented soybean products (jang), kimchi, and jangajji (Korean pickled vegetables), is relatively consistent within Korean dietary culture. In contrast, the intake of other fermented food groups was generally low compared to fermented soybean products and vegetables. However, within specific food groups, such as grains, alcoholic beverages, and dairy products, distinct consumption patterns of fermented foods were observed. The analysis of nutrient intake according to fermented food consumption patterns showed that the LFP group had the lowest intake of energy, carbohydrates, protein, fat, dietary fiber, vitamin A, vitamin C, and potassium compared to the other patterns (Table 4). Nutrient deficiencies can weaken the immune system and increase inflammatory responses, potentially exacerbating the onset and progression of chronic diseases. Such effects may contribute to an increase in hs-CRP and a higher risk of chronic disease development [42]. Therefore, the higher hs-CRP and increased incidence of chronic diseases observed in the LFP group may not be solely due to low fermented food intake but could also be influenced by other contributing factors. Further research is needed to explore these potential influences.

This study has several limitations. First, due to the use of the 24 h recall method for dietary intake assessment, there is a possibility that the reported intake may not accurately reflect habitual consumption. These limitations may lead to the underreporting or overreporting of intake, which can reduce the accuracy of estimated nutrient intake levels and result in the underestimation or overestimation of differences between fermented food consumption patterns. Second, the complex sample design used in the KNHANES was not fully accounted for in the analysis process, which may limit the generalizability of the findings. Lastly, since this study was conducted on a Korean population, the generalizability of the results to other populations may be limited. Despite these limitations, this study is significant in that it classified middle-aged adults into four latent subgroups based on fermented food consumption patterns and analyzed the relationship between hs-CRP and chronic diseases. The findings of this study suggest that fermented food consumption is closely associated with hs-CRP and chronic diseases among middle-aged Korean adults. These results may serve as foundational data for developing health promotion strategies utilizing fermented foods in the future.

## 5. Conclusions

This study classified fermented food consumption patterns and analyzed their relationship with hs-CRP and chronic disease. FGP and FDP showed lower inflammation and reduced odds of hypertension, diabetes, and dyslipidemia compared to LFP. These findings underscore the potential role of fermented food consumption in inflammatory regulation and chronic disease prevention, offering valuable insights for developing personalized dietary guidelines and public health strategies to promote healthier eating habits.

## Figures and Tables

**Table 1:** Latent profile analysis (LPA).

Number of Clusters	AIC	BIC	aBIC	Entropy	Loglike-Lihood	P for LMR	P for BLRT
1	157,404.489	157,473.183	157,441.405	-	−78,692.245	-	-
2	138,978.722	139,122.979	139,056.246	0.965	−69,468.361	0.0000	0.0000
3	127,363.480	127,583.301	127,481.612	0.972	−63,649.740	0.0000	0.0000
4	122,312.456	122,607.840	122,471.196	0.973	−61,113.228	0.0000	0.0000
5	119,808.401	120,179.349	120,007.749	0.969	−59,850.201	0.0000	0.0000

AIC, Akaike information criterion; BIC, Bayesian information criterion; aBIC; adjusted Bayesian information criterion; LMR, Lo–Mendell–Rubin likelihood ratio test; BLRT, bootstrap likelihood ratio test.

**Table 2:** Adjusted mean intake by fermented food group.

Variables	Fermented Food Consumption Patterns			
	Cluster I (n = 3857, 54.2%)	Cluster II (n = 1105, 15.5%)	Cluster III (n = 998, 14.0%)	Cluster IV (n = 1151, 16.2%)
	LFP ^(1)^	FABP ^(2)^	FDP ^(3)^	FGP ^(4)^
Grains	0.000	0.778	0.001	4.097
Fermented soybean products	2.638	2.884	2.538	2.445
Vinegar	0.258	0.381	0.294	0.295
Vegetables	4.422	4.338	4.284	4.131
Fish and seafood	0.401	0.544	0.375	0.365
Fruits	0.009	0.033	0.139	0.040
Dairy products	0.000	0.733	4.100	1.091
Alcoholic beverages	0.024	5.557	0.038	0.023
Sauces	0.009	0.022	0.017	0.023
Leaf teas and beverages	0.000	0.244	0.000	0.000

^(1)^ Low fermented-food pattern; ^(2)^ fermented alcohol- and beverage-centered Pattern; ^(3)^ fermented dairy-centered pattern; ^(4)^ fermented grain-centered pattern.

**Table 3:** General characteristics according to fermented food consumption patterns.

Variables	Fermented Food Consumption Patterns								*p*
	FGP ^(1)^ (n = 1151, 16.2%)		FDP ^(2)^ (n = 998, 14.0%)		FABP ^(3)^ (n = 1105, 15.5%)		LFP ^(4)^ (n = 3857, 54.2%)		
Sex									<0.001
Men (n = 2765)	355	(30.8)	297	(29.8)	565	(51.1)	1548	(40.1)	
Women (n = 4346)	796	(69.2)	701	(70.2)	540	(48.9)	2309	(59.9)	
Age (year)	51.4	±7.1 ^a^	52.7	±7.2 ^b^	50.7	±7.1 ^a^	52.7	±7.1 ^b^	<0.001
BMI (kg/m^2^)	23.0	±2.9 ^a^	23.1	±2.9 ^a^	23.4	±2.9 ^ab^	23.5	±3.0 ^b^	<0.001
Household income									<0.001
Lowest	80	(7.0)	82	(8.2)	84	(7.6)	487	(12.6)	
Lowest middle	235	(20.4)	202	(20.2)	246	(22.3)	932	(24.2)	
Upper middle	347	(30.1)	292	(29.3)	310	(28.1)	1101	(28.5)	
Highest	489	(42.5)	422	(42.3)	465	(42.1)	1337	(34.7)	
Marital status									<0.001
Yes	1113	(96.7)	967	(96.9)	1066	(96.5)	3650	(94.6)	
No	38	(3.3)	31	(3.1)	39	(3.5)	207	(5.4)	
Alcohol consumption									<0.001
Yes	519	(45.1)	432	(43.3)	938	(84.9)	1978	(51.3)	
No	632	(54.9)	566	(56.7)	167	(15.1)	1879	(48.7)	
Smoking									<0.001
Yes	136	(11.8)	106	(10.6)	288	(20.6)	763	(19.8)	
No	1015	(88.2)	892	(89.4)	877	(79.4)	3094	(80.2)	
Aerobic activity									0.033
Yes	95	(8.3)	64	(6.4)	89	(8.1)	240	(6.2)	
No	1056	(91.7)	934	(93.6)	1016	(91.9)	3617	(93.8)	
WC (cm)	79.4	±8.9 ^a^	79.3	±8.6 ^a^	81.3	±8.6 ^b^	81.2	±8.9 ^b^	<0.001
SBP (mmHg)	116.2	±15.9 ^a^	116.5	±15.3 ^a^	118.4	±15.8 ^b^	118.4	±16.1 ^b^	<0.001
DBP (mmHg)	76.3	±9.6 ^a^	76.5	±9.5 ^a^	78.5	±10.0 ^b^	77.2	±9.8 ^a^	<0.001
FBG (mg/dL)	98.7	±22.7 ^a^	98.4	±19.5 ^a^	102.0	±26.3 ^b^	102.3	±26.2 ^b^	<0.001
HbA1c (%)	5.7	±0.7 ^ab^	5.6	±0.6 ^a^	5.6	±0.8 ^a^	5.7	±0.8 ^b^	<0.001
HDL-cholesterol (mg/dL)	53.4	±12.9 ^b^	52.6	±12.6 ^b^	53.8	±13.6 ^b^	51.1	±12.9 ^a^	<0.001
LDL-cholesterol (mg/dL)	120.8	±33.0 ^b^	120.2	±34.2 ^b^	114.9	±35.1 ^a^	117.4	±36.0 ^ab^	<0.001
TG (mg/dL)	130.4	±106.6 ^a^	124.3	±94.2 ^a^	143.2	±118.7 ^b^	144.7	±123.1 ^b^	<0.001
Total cholesterol	200.3	±35.9 ^a^	197.7	±36.9 ^a^	197.3	±36.7 ^a^	197.4	±36.6 ^a^	0.111
hs-CRP (mg/L)	0.9	±1.2 ^ab^	0.9	±1.2 ^ab^	0.8	±1.1 ^a^	1.0	±1.2 ^b^	<0.001

Values are presented as mean ± SE or n (%). Post hoc comparisons were performed using the Scheffé test. Superscript letters (a, b) indicate statistically significant differences between groups based on post-hoc comparisons (*p* < 0.05). Groups that do not share the same letter are significantly different. BMI, body mass index; SBP, systolic blood pressure; DBP, diastolic blood pressure; FBG, fasting blood glucose; HbA1c, glycated hemoglobin; HDL, high-density lipoprotein; LDL, low-density lipoprotein; TG, triglyceride; hs-CRP, high-sensitivity C-reactive protein; ^(1)^ fermented grain-centered pattern; ^(2)^ fermented dairy-centered pattern; ^(3)^ fermented alcohol- and beverage-centered pattern; ^(4)^ low fermented-food pattern.

**Table 4:** Nutrition intake by fermented food consumption patterns.

Variables	Fermented Food Consumption Patterns								*p*
	FGP ^(1)^ (n = 1151, 16.2%)		FDP ^(2)^ (n = 998, 14.0%)		FABP ^(3)^ (n = 1105, 15.5%)		LFP ^(4)^ (n = 3857, 54.2%)		
Energy (kcal/day)	2032.1	±759.2 ^c^	1947.1	±704.6 ^b^	2407.8	±905.9 ^d^	1846.7	±732.6 ^a^	<0.001
Carbohydrate (g)	323.6	±123.9 ^b^	311.7	116.4 ^b^	314.6	124.4 ^b^	297.1	±118.7 ^a^	<0.001
Protein (g)	70.8	±33.5 ^b^	70.3	±31.5 ^b^	86.1	±38.1 ^c^	64.4	±31.5 ^a^	<0.001
Fat (g)	47.0	±28.1 ^c^	43.5	±27.3 ^b^	50.7	±30.5 ^d^	35.9	±25.6 ^a^	<0.001
CHO/PRO/FAT (%)	73.2:16.1:10.7		73.2:16.6:10.2		69.5:19.2:11.2		74.7:16.3:9.0		-
Dietary fiber (g)	29.2	±15.5 ^b^	29.2	±13.3 ^b^	27.8	±14.5 ^ab^	26.7	±14.5 ^a^	<0.001
Vitamin A (µg RAE)	678.8	±657.9 ^ab^	747.6	±841.9 ^bc^	790.8	±995.3 ^c^	647.7	±727.8 ^a^	<0.001
Vitamin C (mg)	85.9	±98.4 ^a^	90.2	±91.3 ^a^	85.0	±162.8 ^a^	79.4	±91.6 ^a^	0.018
Sodium (mg)	3403.5	±1914.9 ^a^	3354.4	±1916.2 ^a^	4156.3	±2493.6 ^b^	3482.3	±2102.5 ^a^	<0.001
Potassium (mg)	3093.8	±1437.0 ^a^	3299.6	±1544.2 ^b^	3387.7	±1422.0 ^b^	3482.3	±2102.5 ^a^	<0.001
Cholesterol (mg)	256.2	±225.6 ^c^	242.2	±222.6 ^b^	306.4	±236.1 ^b^	202.1	±195.3 ^a^	<0.001

Values are presented as mean ± SE. Post hoc comparisons were performed using the Scheffé test. Superscript letters (a, b, c, d) indicate statistically significant differences between groups based on post-hoc comparisons (*p* < 0.05). Groups that do not share the same letter are significantly different. CHO, carbohydrate; PRO, protein; RAE, retinol activity equivalent; ^(1)^ fermented grain-centered pattern; ^(2)^ fermented dairy-centered pattern; ^(3)^ fermented alcohol- and beverage-centered pattern; ^(4)^ low fermented-food Pattern.

**Table 5:** Relationship between fermented food consumption patterns and hs-CRP.

Variables	Fermented Food Consumption Patterns								*p*
	FGP ^(1)^ (n = 1151, 16.2%)		FDP ^(2)^ (n = 998, 14.0%)		FABP ^(3)^ (n = 1105, 15.5%)		LFP ^(4)^ (n = 3857, 54.2%)		
hs-CRP									0.003
1 mg/L	884	(76.8)	788	(79.0)	867	(78.5)	2844	(73.7)	
1–3 mg/L	199	(17.3)	161	(16.1)	183	(16.6)	761	(19.7)	
3 mg/L	68	(5.9)	49	(4.9)	55	(5.0)	252	(6.5)	

hs-CRP, high-sensitivity C-reactive protein; ^(1)^ fermented grain-centered pattern; ^(2)^ fermented dairy-centered Pattern; ^(3)^ fermented alcohol- and beverage-Centered Pattern; ^(4)^ low fermented-food pattern.

**Table 6:** OR Analysis of hs-CRP by fermented food consumption patterns.

Variables	Fermented Food Consumption Patterns				*p*
	Cluster IV ^(1)^	Cluster III ^(2)^	Cluster II ^(3)^	Cluster I ^(4)^	
hs-CRP 1–3 mg/L ^(5)^ (n = 6687)					
Crude ORs (95% CIs)	0.841 (0.707–1.001)	0.764 (0.633–0.921)	0.789 (0.660–0.943)	1.0 (ref)	<0.001
Model I ^(7)^ ORs (95% CIs)	0.902 (0.757–1.075)	0.804 (0.665–0.971)	0.837 (0.696–1.008)	1.0 (ref)	<0.001
Model II ^(8)^ ORs (95% CIs)	0.975 (0.849–1.120)	0.852 (0.736–0.986)	0.807 (0.700–0.931)	1.0 (ref)	<0.001
Model III ^(9)^ ORs (95% CIs)	0.805 (0.668–0.971)	0.822 (0.679–0.994)	0.918 (0.769–1.096)	1.0 (ref)	<0.001

hs-CRP ≥ 3 mg/L ^(6)^ (n = 5807)					
Crude ORs (95% CIs)	0.868 (0.657–1.147)	0.702 (0.512–0.963)	0.716 (0.530–0.968)	1.0 (ref)	<0.001
Model I ^(7)^ ORs (95% CIs)	0.967 (0.730–1.281)	0.762 (0.526–1.048)	0.718 (0.526–0.980)	1.0 (ref)	<0.001
Model II ^(8)^ ORs (95% CIs)	0.975 (0.736–1.293)	0.769 (0.559–1.058)	0.735 (0.541–0.999)	1.0 (ref)	<0.001
Model III ^(9)^ ORs (95% CIs)	0.952 (0.717–1.265)	0.758 (0.551–1.044)	0.708 (0.517–0.970)	1.0 (ref)	<0.001

OR, odds ratio; CI, confidence interval; ^(1)^ fermented grain-centered pattern; ^(2)^ fermented dairy-centered pattern; ^(3)^ fermented alcohol- and beverage-centered pattern; ^(4)^ low fermented-food pattern; ^(5)^ ref: <1 mg/L, comparison: 1–3 mg/L; ^(6)^ ref: <1 mg/L, comparison: ≥3 mg/L; ^(7)^ tests were adjusted for sex, age, smoking, and drinking; ^(8)^ tests were adjusted for sex, age, smoking, drinking, and marital, income; ^(9)^ tests were adjusted for sex, age, smoking, drinking, marital, income, physical activity, and energy.

**Table 7:** OR Analysis of chronic diseases by fermented food consumption patterns.

Variables	Fermented Food Consumption Patterns				*p*
	Cluster IV ^(1)^	Cluster III ^(2)^	Cluster II ^(3)^	Cluster I ^(4)^	
Hypertension					
Crude ORs (95% CIs)	0.697 (0.598–0.813)	0.813 (0.694–0.951)	0.941 (0.812–1.091)	1.0 (ref)	<0.001
Model I ^(5)^ ORs (95% CIs)	0.810 (0.690–0.950)	0.863 (0.732–1.017)	0.971 (0.829–1.139)	1.0 (ref)	<0.001
Model II ^(6)^ ORs (95% CIs)	0.826 (0.704–0.970)	0.881 (0.747–1.038)	1.070 (0.915–1.252)	1.0 (ref)	<0.001
Model III ^(7)^ ORs (95% CIs)	0.831 (0.707–0.976)	0.885 (0.750–1.044)	1.088 (0.926–1.278)	1.0 (ref)	<0.001
Diabetes					
Crude ORs (95% CIs)	0.506 (0.399–0.642)	0.583 (0.459–0.742)	0.728 (0.588–0.901)	1.0 (ref)	<0.001
Model I ^(5)^ ORs (95% CIs)	0.586 (0.459–0.747)	0.621 (0.486–0.794)	0.760 (0.606–0.951)	1.0 (ref)	<0.001
Model II ^(6)^ ORs (95% CIs)	0.608 (0.476–0.775)	0.646 (0.505–0.827)	0.795 (0.636–0.993)	1.0 (ref)	<0.001
Model III ^(7)^ ORs (95% CIs)	0.607 (0.475–0.776)	0.647 (0.506–0.829)	0.799 (0.636–1.005)	1.0 (ref)	<0.001
Dyslipidemia					
Crude ORs (95% CIs)	0.826 (0.724–0.943)	0.778 (0.676–0.896)	0.751 (0.656–0.860)	1.0 (ref)	<0.001
Adjusted I ^(5)^ ORs (95% CIs)	0.950 (0.827–1.090)	0.832 (0.719–0.962)	0.832 (0.720–0.962)	1.0 (ref)	<0.001
Adjusted II ^(6)^ ORs (95% CIs)	0.975 (0.849–1.120)	0.852 (0.736–0.986)	0.807 (0.700–0.931)	1.0 (ref)	<0.001
Adjusted III ^(7)^ ORs (95% CIs)	0.988 (0.859–1.135)	0.858 (0.741–0.994)	0.826 (0.714–0.957)	1.0 (ref)	<0.001

OR, odds ratio; CI, confidence interval; ^(1)^ fermented grain-centered pattern; ^(2)^ fermented dairy-centered pattern; ^(3)^ fermented alcohol- and beverage-centered Pattern; ^(4)^ low fermented-food pattern; ^(5)^ tests were adjusted for sex, age, smoking, and drinking; ^(6)^ tests were adjusted for sex, age, smoking, drinking, marital status, and income; ^(7)^ Tests were adjusted for sex, age, smoking, drinking, marital status, income, physical activity, and energy.

## Data Availability

The original contributions presented in this study are included in the article/Supplementary Material. Further inquiries can be directed to the corresponding author.

## Supplementary Materials

The following supporting information can be downloaded at: https://www.mdpi.com/article/10.3390/nu17081343/s1, Table S1: Fermented food groups and items.

## Abbreviations

hs-CRP	High-sensitivity C-reactive protein
KNHANES	Korea National Health and Nutrition Examination Survey
LPA	Latent profile analysis
ADA	American Diabetes Association
KSH	Korean Society of Hypertension
NCEP	National Cholesterol Education Program
ATP III	Adult Treatment Panel III
OR	Odds ratio
CI	Confidence interval
LFP	Low fermented-food pattern
FABP	Fermented alcohol-and beverage-centered pattern
FDP	Fermented dairy-centered pattern
FGP	Fermented grain centered pattern
TG	Triglyceride
HDL	High-density lipoprotein
LDL	Low-density lipoprotein
HbA1c	Glycated hemoglobin
FBG	Fasting blood glucose
BMI	Body mass index
ZIP	Zero-inflated Poisson
AIC	Akaike information criterion
BIC	Bayesian information criterion
aBIC	Adjusted Bayesian information criterion
LMR	Lo–Mendell–Rubin
BLRT	Bootstrap likelihood ratio test
LMR-LRT	Lo–Mendell–Rubin likelihood ratio test
