# Supplementary material for: Relationship Between Fermented Food Consumption Patterns, hs-CRP, and Chronic Diseases Among Middle-Aged Koreans: Data from the 2015–2018 Korea National Health and Nutrition Examination
Source: Nutrients. 2025 Apr 14;17(8):1343. doi: 10.3390/nu17081343 (PMC12030273; doi:10.3390/nu17081343)
Supplement: Supplementary file 1 [file nutrients-17-01343-s001.zip › nutrients-3518595-supplementary.pdf]

Table S1. Fermented Food Groups and Items

Food Groups	Food Items
Grains	baguette, sourdough bread, steamed bun, bagel, steamed fermented rice cake, nann, sikhye(korean sweet rice beverage), jocheong(korean rice syrup)
Fermented Soybean products	ganjang(soy sauce), doenjang(fermented soybean paste), gochjang(fermented red pepper soybean paste), cheonggukjang(fast-fermented soybean paste), natto(Japanese fermented whole soybean paste), ssamjang(a mixture of gochujang and doenjang), chunjang(fermented black soybean paste)
Vinegar	vinegar, vinegar-based beverage, fruit vinegar
Vegetables	danmuji (yellow pickled radish), jangajji(korean pickled vegetables), kimchi
Fish and Seafood	jeotgal(fermented seafood), aekjeot(fermented fish sauce), fermented fish
Fruits	pickled plums, pickled olives
Dairy products	liquid yogurt, yogurt, cheese
Alcoholic Beverages	beer, wine, makgeolli(korean rice wine), fruit wine, cheongju(korean refined rice wine)
Sauces	balsamic dressing, hot sauce, fish sauce
Leaf Teas and Beverages	fermented tea, fermented beverage
